# Study of Lignin Extracted from Rubberwood Using Microwave Assisted Technology for Fuel Additive

**DOI:** 10.3390/polym14040814

**Published:** 2022-02-20

**Authors:** Trakarn Yimtrakarn, Watchareeya Kaveevivitchai, Wen-Chien Lee, Nuttapol Lerkkasemsan

**Affiliations:** 1 Department of Chemical Engineering, School of Engineering, King Mongkut’s Institute of Technology Ladkrabang, Ladkrabang, Bangkok 10520, Thailand; 63601052@kmitl.ac.th; 2 Department of Chemical Engineering, National Cheng Kung University, Tainan City 70101, Taiwan; wkaveechai@mail.ncku.edu.tw; 3 Department of Chemical Engineering, Systems Biology and Tissue Engineering Research Center, National Chung Cheng University, Chiayi 621, Taiwan; chmwcl@ccu.edu.tw

**Keywords:** delignification, biomass deconstruction, microwave

## Abstract

Lignin is the most abundant natural aromatic polymer, especially in plant biomass. Lignin-derived phenolic compounds can be processed into high-value liquid fuel. This study aimed to determine the yield of lignin by the microwave-assisted solvent extraction method and to characterize some essential properties of the extracted lignin. Rubberwood sawdust (*Hevea brasiliensis*) was extracted for lignin with an organic-based solvent, either ethanol or isopropanol, in a microwave oven operating at 2450 MHz. Two levels of power of microwave, 100 W and 200 W, were tested as well as five extraction times (5, 10, 15, 20, 25, and 30 min). The extracted lignin was characterized by Klason lignin, Fourier transform infrared spectroscopy (FT-IR), 2D HSQC NMR, Ultraviolet-visible spectrophotometry (UV-vis), and Bomb calorimeter. The results showed that the yield of extracted lignin increased with the extraction time and power of the microwave. In addition, the extraction yield with ethanol was higher than the yield with isopropanol. The highest yield was 6.26 wt.%, with ethanol, 30 min extraction time, and 200 W microwave power.

## 1. Introduction

Industrial technology is rapidly depleting limited fossil fuels. For this reason, renewable energy is becoming an important alternative [1]. Lignin has received a lot of attention for its various applications, for example, dispersant [2,3,4], protective UV-absorbent [5,6], (nano)composite [7,8], and nanoparticle [9,10]. In particular, there is valorization of lignin as a value added product, a lignin nanoparticle can transform to highly fluorescent graphene quantum dots [11]. Due to the complexity of the lignin structure, the pretreatment process to extract lignin from biomass is an important part of the whole extraction process. Usage of a lignin extracted by a particular method requires characterization for it to suit a particular application [12]. Currently, numerous technologies for producing lignin have received increasing attention, such as supercritical solvent extraction, hydrogenolysis, oxidation, acid hydrotropic fractionation, and solvolysis [13,14,15,16,17].

Lignin extraction from biomass can be carried out by a chemical delignification method, using either aqueous or organic solvent [18]. Alcohols are the most widely used organic solvent for delignification [19,20]. Ethanol and isopropanol were selected as the extraction solvents in this experiment because they were eco-friendly, less hazardous, easy to recover, and easily diffused into the wood [21]. A previous study by Chutikan Inkrod et al. [22] also extracted lignin from low-cost Para rubberwood sawdust like ours and achieved a 20% extraction efficiency at 160 °C with a ternary solvent (MBK, ethanol, and water) without any catalyst. Another group of researchers, Ahmad Adlie Shamsuri and Dzulkefly Kuang Abdullah, successfully extracted lignin from rubberwood by ionic liquid. The optimum conditions were ionic liquid 0.5 moles, 120 min of extraction time, and 100 °C temperature, which provided 13.03 wt.% yield.

Microwave technology is an alternative biomass heating method. Microwave radiates electromagnetic energy directly generates heat inside a material. In contrast, other methods generate heat by conduction. This technique can reduce extraction time, increase the yield of a product, save energy, and create uniform heat distribution [23]. The use of microwave and ethanol to successfully extract polyphenol from grape peel by YU Hai-bo et al. [24] was similar to our use of ethanol and microwave to extract lignin from sawdust. Some of the benefits of that technique were shorter extraction time, prevention of polyphenol oxidation, and increasing polyphenol yield, which we would also like our technique to provide. The benefit of shorter extraction time when the extraction is assisted by microwave was also reported by Saksit Imman et al. [25]. The team extracted lignin contained in rice straw by microwave-assisted solvolysis method and found that microwave treatment appeared to provide a higher reaction rate and shorter reaction time with more uniform heat distribution. Long Zhou et al. [26] concluded that the microwave heating method receives a higher yield and purity of lignin compared with a conventional method. That conclusion was also supported by a previous study [27].

This study investigated the extraction of lignin from sawwood dust by microwave-assisted solvolysis with ethanol and propanol. As a part of the investigation, the extracted lignin from rubberwood was characterized. The ultimate goal for this investigation was to replace difficult-to-recover acidic or alkaline solvents with ethanol or propanol that were much safer and easier to recover.

## 2. Materials and Methods

### 2.1. Materials

Rubberwood sawdust was provided by a local wood mill in (Sri Incense (Thailand) Co., Ltd., Bangkok, Thailand) Thailand. Rubberwood sawdust was kept in the oven at 105 °C for 24 h for drying. Then, the material was kept in a beaker with a plastic seal to avoid moisture until needed for the experiment. Ethanol (95%) and isopropanol (99.8%) were from Chemipan, Bangkok, Thailand. Sulfuric acid (95–97%) was obtained from Merck (Bankok, Thailand). 

### 2.2. Solvent Extraction with Microwave-Assisted Method

**Figure 1:**
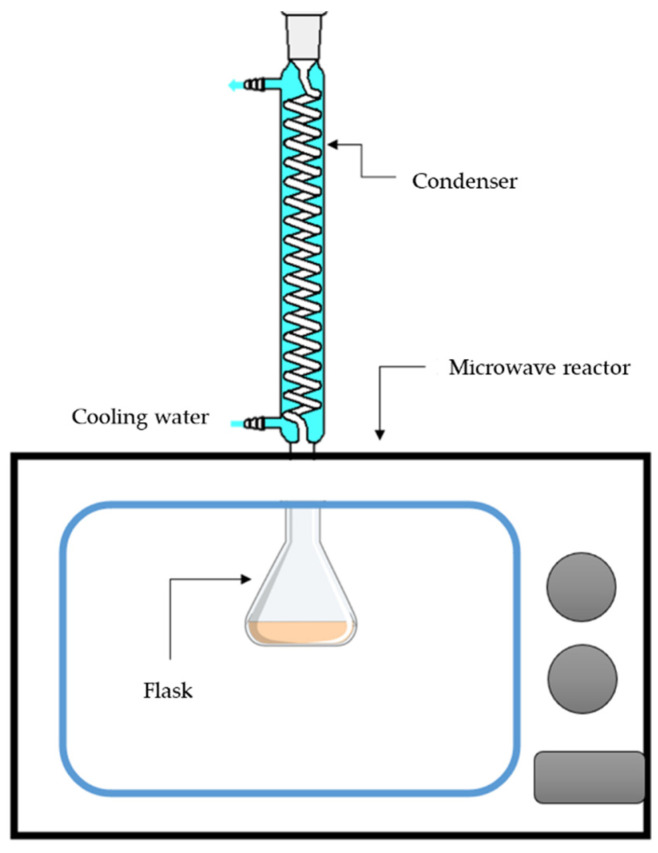
Schematic of the modified microwave oven.

The lignin extraction from the biomass process was carried out in a 500 mL Erlenmeyer flask. The dried rubberwood sawdust 5 g (solid-to-liquid ratio of 1:10 by weight) was mixed with ethanol or isopropanol prepared in room conditions. The flask was shaken and set up with a modified microwave (SAMSUNG GE711K/XST Microwave, Thai Samsung Electronics Co., Ltd., Bangkok, Thailand) (750 W, 20 L, 2450 MHz)) (Figure 1). The condenser was closed with a plastic seal. The solvent extraction was performed in the microwave oven at the power of microwave (100 W and 200 W) and extraction time (5–30 min with 5 min increments). Then, the solid fraction was separated from the mixture by vacuum filtration. The solid residue was dried in the oven at 105 °C within the Petri dish. The liquid solution was collected for further characterization and quantitation.

### 2.3. Klason Lignin Analytical Method

Then, 1 g of dried wood was hydrolyzed by 15 mL of 72% sulfuric acid and stirred with a glass rod. After the specimen dispersion, the beaker was covered with a watch glass and kept in a water bath for 2 h. Then, 300–400 mL of water was added into a 1000 mL flask. The material in the beaker was conducted to the flask. The solution was diluted by rinse water to fill a total volume of 575 mL. The solution was boiled in the reflux system to maintain its volume for 4 h. Lignin was precipitated by keeping the flask at the inclined position for 12 h. Then, the solution was filtrated by vacuum filtration and washed acid remaining in precipitated lignin by using hot water. The filtration paper with lignin was kept in the oven at 105 °C for 24 h [28]. Finally, the lignin in the dried wood (Klason lignin) was calculated by Equation (1):

Lignin % = *A* × 100/*W* (1)

where

Lignin % = lignin content in the wood sample
*A* = weight of lignin, g
*W* = dry weight of test specimen, g

### 2.4. Ultraviolet-Visible Spectrophotometry (UV-vis) Analytical Method

In the UV-visible spectrophotometer analysis, the solution samples were analyzed using a UV-visible spectrophotometer (Thermo Evolution 201, Madison, WI, USA). First, 0.3 mL of lignin sample was dissolved in 2.7 mL 95% *v*/*v* ethanol in a quartz cuvette. Then, the sample absorbance was recorded at the wavelength of 200–400 nm. The weight of extracted lignin was determined by a UV-visible spectrophotometer compared with a standard curve. The yield of extracted lignin was compared with the total lignin contained in rubberwood from the Klason method (0.02064 g of lignin/g of rubberwood sawdust).

The yield percentage of extracted lignin is calculated by Equation (2):

(2) $$Yield_{Lignin}=\frac{W_{ExtractedLignin}}{W_{TotalLignin}}\times 100\%$$

where

*W_ExtractedLignin_* = weight of lignin extracted, g
*W_TotalLignin_* = weight of lignin present in the feedstock, g

### 2.5. Fourier Transform Infrared Spectroscopy (FT-IR) Characterization Method

The prepared sample was inspected by the ATR technique. FT-IR data that were analyzed from Sci-Ins, King Mongkut’s Institute of Technology, Ladkrabang, were measured on the FT-IR Spectrometer (Nicolet iS20, Madison, WI, USA). The data were recorded with a 4 cm^−1^ spectral resolution and a range of 4000 to 700 cm^−1^.

### 2.6. 2D HSQC NMR Characterization Method

An amount of either 40 mg or 0.6 mL of all samples was dissolved in 0.5 mL of methanol-d_4_. NMR spectra were collected by NMR spectroscopy (JEOL JNM-ECZR 500 MHz, Tokyo, Japan), which was analyzed from Sci-Ins, King Mongkut’s Institute of Technology, Ladkrabang. The spectral widths were 6 ppm and 100 ppm for the ^1^H- and ^13^C-dimensions, respectively. A dimension size consisting of 256 × 819 points was obtained in 8 scans with a 0.164 s acquisition time and 1 s relaxation delay. The ^1^J_CH_ used constantly was 145 Hz.

### 2.7. Bomb Calorimeter Characterization Method

For the Bomb calorimeter analysis, 20–50 mL of extracted solution was prepared in a stainless-steel vessel with oxygen supplied. The vessel containing the solution sample was inserted into the calorimeter, with temperatures ranging from 18 °C to 24 °C. The higher heating value (HHV) that was analyzed from STREC, Chulalongkorn University was measured on the LECO AC500 Isoperibol calorimeter. 

## 3. Results and Discussion

### 3.1. Microwave-Assisted Extraction of Lignin

**Figure 2:**
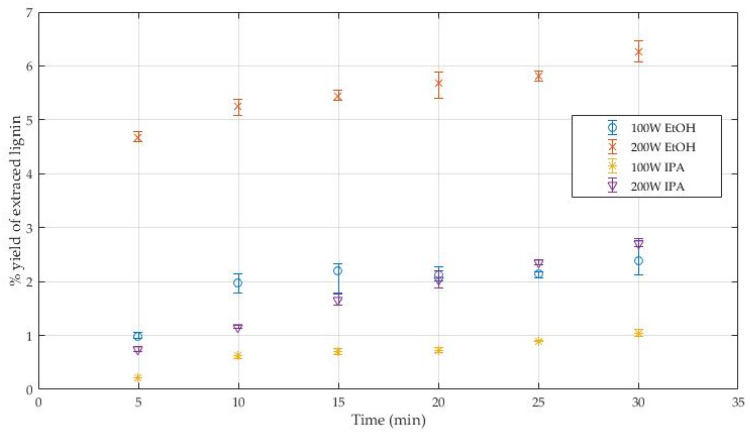
Percent yield of extracted lignin.

Figure 2 illustrates the percent yield of lignin recovered from deconstructed biomass in a microwave oven using various solvents and microwave power parameters. When isopropanol was used as the solvent, the lowest percent yield of extracted lignin was observed. Lignin has a poor capacity for solubility in isopropanol. With a faster rate of delignification, ethanol is more effective than isopropanol. Even at 100 W, ethanol had more extracted lignin, because primary alcohols are more selective than secondary and ternary alcohols in delignification [29]. Furthermore, ethanol has a lower molecular weight, which leads to greater permeability and fluidity [19]. The hydroxyl group in alcohols has nucleophilic reactive capabilities. The glycosidic bond in cellulose and the ether bond in lignin can both be decomposed by alcohol [30]. The highest lignin concentration was found when the microwave power was set to 200 W for 30 min, and ethanol was used as the solvent. Furthermore, isopropanol produced a poor yield of extracted lignin, with a maximum yield of only 2.72 percent, whereas ethanol produced a yield of 6.26 percent under the same conditions. Although isopropanol may reach a higher maximum temperature in the microwave than ethanol, its solubility capacity has a greater impact.

The operational power is a direct temperature response. Microwave power influences a solvent’s boiling point. Low-boiling-point solvents include ethanol and isopropanol. The boiling point of water can exceed that of the atmosphere during microwave extraction [31]. The boiling point of ethanol 20 mL reached roughly 12 kelvins during microwave operation at 100 W, according to Farid Chemat and Erik Esveld [31]. The solubility of most substances increases as the temperature rises. As microwave power is increased, lignin solubility in the solvent increases. Furthermore, increasing the temperature reduces the viscosity of the alcohol solvent and improves its diffusivity. It encourages effective lignocellulose-solvent interaction. As a result, increasing the microwave power increases the lignin yields [32]. Microwave power was increased from 100 W to 200 W, which resulted in a lignin production from 2.38 percent to 6.26 percent in ethanol solvent after 30 min. At the same duration, the yield of lignin in isopropanol increased from 1.05 to 2.72 percent. The effect of extracted duration is that at short, extracted times (5 min), the yield of lignin is low because the reaction temperature is low. This time is insufficient to break the strong bond between lignin and the other component. As a result, with increasing time, lignin yield and biomass depolymerization efficiency improve. This method less extracted lignin than the organosolv method. Nevertheless, the microwave-assisted method in this method cannot be operated with a higher power due to the solvent evaporating too rapidly to reflux. However, using the organosolv technique to remove lignin results in waste from the acid or alkaline catalyst. The absence of acid or alkaline catalysts during processing is a benefit of another technique.

### 3.2. FT-IR Analytical Method

**Figure 3:**
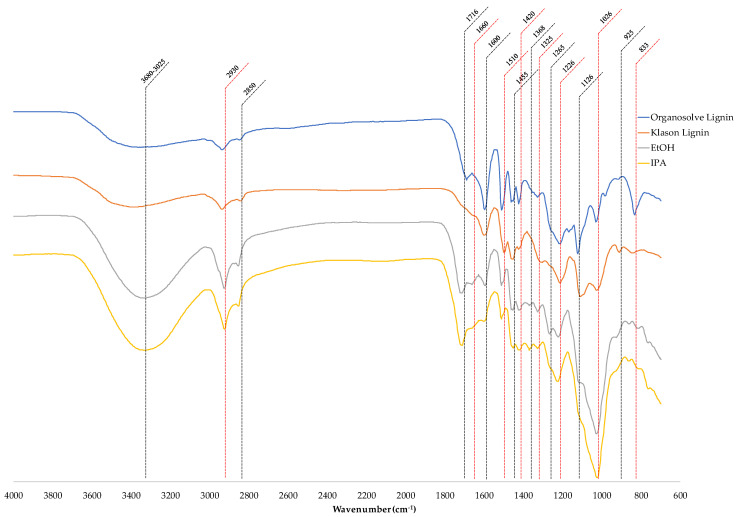
FT-IR spectra of lignin by organosolv, Klason, and solvent extraction with the microwave-assisted method.

The complete results are shown in Figure 3 and Table 1. The FT-IR spectra of products are compared with the commercial organosolv lignin and Klason lignin from rubberwood.

FT-IR results, the spectra of extracted lignins that are extracted by isopropanol and ethanol, had no significant difference. In addition, the Klason and organosolv method were not the same as the sample from this extraction. The board band at 3680–3025 cm^−1^ was the O-H stretching vibration in the aliphatic and aromatic hydroxy groups. At 3680–3025 cm^−1^, the extracted lignin using isopropanol as solvent achieved higher relative intensities indicating the higher hydroxy group content in the solution. Moreover, the lignin from Klason and organosolv that used acid as a catalyst had lower oxidation compared with this method. The bands at 2930 cm^−1^ and 2850 cm^−1^ were CH stretch in the methyl and methylene group and C–H stretch in O–CH_3_ group, respectively. The peak at 1716 cm^−1^ was a C=O stretch of unconjugated carboxyl, ketone, and ester groups, which could be used to investigate the lignin and remaining hemicellulose during the extraction [25,33]. The peak at 1660 cm^−1^ was assigned to the conjugated carbonyl stretching in lignin. The peaks at 1600, 1510, 1170, and 1420 cm^−1^ were aromatic skeletal vibrations, and the C-H deformation frequencies combined with aromatic ring vibration at 1455 cm^−1^. The peaks in the Klason and organosolv lignin showed a clearer peak because these methods have precipitation of lignin in the water. In contrast, this method must be aware of moisture content in the solution. The phenolic hydroxyl group was represented by the absorbance at 1368 cm^−1^, which can be attributed to the breaking of β-O-4 bonds during extraction. This band indicated that the microwave irritation not only broke the bond connecting lignin with cellulose and hemicellulose but also broke the ether bond in the lignin [34]. The peaks at 1265, 1226, and 833 cm^−1^ approached guaiacyl units. The guaiacyl (coniferyl alcohol) is the highest monomer content in lignin from rubberwood. The absorbance at 1325 and 1126 cm^−1^ was the syringyl ring breathing and C–O stretching. The absorbance at 1026 cm^−1^ was assigned to the C–O–C mode of the glycosidic linkages [33]. This absorbance at 1026 cm^−1^ indicated that glycosidic of cellulose and hemicellulose remained in the solution. At 925 cm^−1^ a pyranose ring in hemicellulose was indicated. The solutions from both solvent extractions were rich in phenolic, carbonyl, and aromatic compounds. The spectra identified that the samples were composed of lignin. However, the samples were contaminated with cellulose and hemicellulose that remained during extraction.

### 3.3. 2D HSQC NMR Analytical Method

**Figure 4:**
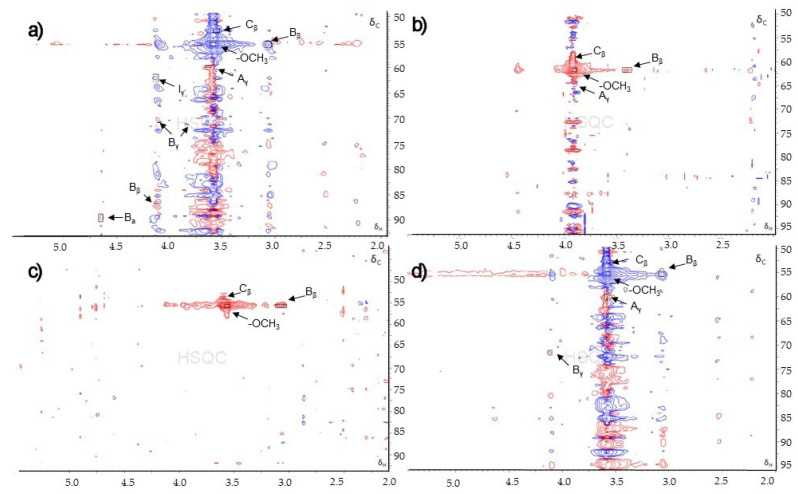
HSQC spectra (δ_C_/δ_H_ 50–95/2.0–5.5) of (**a**) solutions of extracted lignin by using ethanol, (**b**) solutions of extracted lignin using isopropanol, (**c**) Klason lignin, and (**d**) Organosolv lignin.

The two-dimensional ^13^C-^1^H HSQC association spectrum indicated carbons attached with protons, which is a useful method for understanding the lignin structure. The extracted lignins, Klason lignin, and organosolv lignin were subjected to 2D NMR analysis. The HSQC spectra showed a region corresponding to the side chain region. The side chain regions (δ_C_ 50–95 ppm and δ_H_ 2.0–5.5 ppm) are shown in Figure 4. The main spectra were indicated in the HSQC spectra by comparison with previous literature data [37,38,39] and are exhibited in Table 2. 

The internal linkages in the lignin structure were present in the side chain region of the HSQC spectra. All of the lignin samples showed the most predominant, which was the methoxyls (δ_C_/δ_H_ 55.48/3.06) linkage. The resinol substructures (B) were detected by their C-H correlation for C_β_−H_β,_ C_γ_−H_γ,_ and C_a_−H_a_ at δ_C_/δ_H_ 55.48/3.06, 70.79/4.10 & 71.21/3.82, and 89.47/4.65. The extracted lignin using ethanol as solvent showed a high content of methoxyls and resinol substructure. β-O-4 substructures (A) were confirmed by C-H correlations at δ_C_/δ_H_ 59.83/3.63 and 86.52/4.12 for C_γ_−H_γ_ and C_β_−H_β_. Phenylcoumaran substructures (C) and cinnamyl alcohol end-groups (I) presented some C-H correlation at δ_C_/δ_H_ 52.81/3.55 and 62.08/4.13, respectively. However, all correlations were detected at a low contour level except methoxyls correlation, which had a strong spectrum. For this reason, it is difficult to indicate the C-H correlation of the lignin structure due to a lot of undesired peaks.

### 3.4. UV-Vis Analytical Method

**Figure 5:**
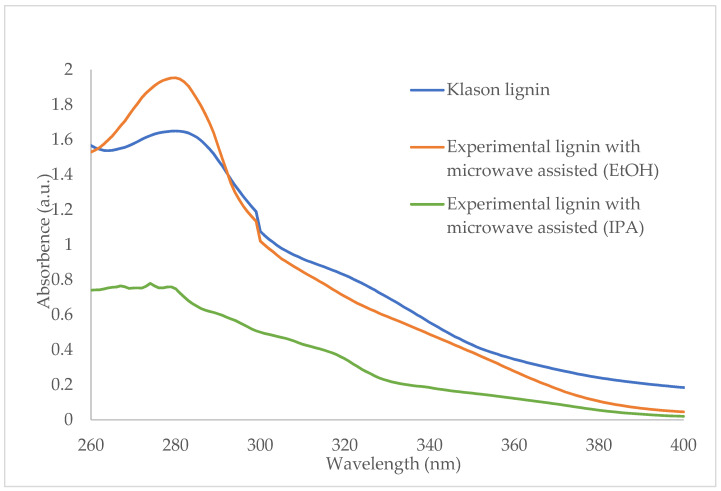
UV-visible spectra of experimental lignins and Klason lignin.

The UV spectra of products are shown in Figure 5. Lignin solutions presented UV spectra characteristic of lignin with the maximal peak at 280 nm, which indicated the non-conjugated phenolic in lignin [40,41]. The Klason lignin was used for comparing with the lignin solution from the experimental method. The extracted lignin solutions from both solvents showed a maximum peak at 280 nm, the same as the Klason lignin. In addition, the absorbance of the extracted lignin by isopropanol was lower than ethanol as solvent at the same conditions. This result indicated the ethanol solvent had high extract efficiency. In the solution extracted by isopropanol, the absorbance at 208 indicated isopropanol absorption.

### 3.5. HHV Characterization Method

**Figure 6:**
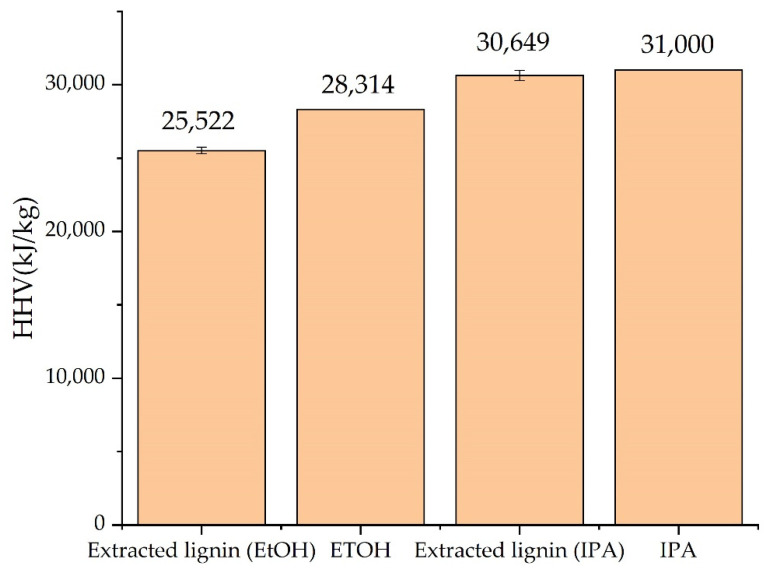
HHV of solutions of the extracted lignins and their solvent.

Figure 6 shows the HHV of the extracted solution compared with pure solvent (95.6% *v*/*v* ethanol and 99.8% *v*/*v* isopropanol). The extraction condition of solutions of extracted lignin was selected, which was optimized at a microwave power of 200 W and 30 min. HHV of both solutions of extracted lignin were lower than the pure solvent. HHV of lignin (17,000–25,000 kJ/kg) depends on the molecular weight of lignin [42]. In addition, the heating value of lignin was lower than the pure alcohol solvent. The results showed that the extracted lignin that contained lignin and alcohol had lower HHV than the pure alcohol solvent. The HHV of the solutions of extracted lignin by using ethanol as a solvent was less than 9.86% compared with ethanol 95%. The HHV of the solutions of extracted lignin by using isopropanol as a solvent was less than 1.13% compared with isopropanol. Several works have used lignin as an additive in transport fuel [43,44]. The extracted solution could be an additive in gasoline and/or diesel rich of in hydroxyl, phenolic, carbonyl, and aromatic compounds. However, this extracted lignin must be tested for quality and performance, such as combustion performance tests, engine efficiency tests, emission tests, etc.

## 4. Conclusions

The objective of this research was to investigate lignin extraction using a solvent extraction method with a microwave-assisted method. According to the results, ethanol extraction was more effective than isopropanol in terms of lignin solubility. With increasing microwave operating time and power, the yield of extracted lignin recovery improved. At 200 W for 30 min and ethanol as the solvent, the highest lignin recovery yield was 6.26 percent. The link between lignin and cellulose and hemicellulose was broken by microwave irritation. Microwave breaks the ether link that connects the lignin monomer. Furthermore, because this technique did not involve lignin precipitation in water, the extracted lignin was contaminated with hemicellulose and cellulose. Because lignin has a low heating value, the heating value of the extracted solutions was lower than that of the pure solvent. The extracted solution could be an additive in fuel rich in hydroxyl, phenolic, carbonyl, and aromatic compounds.

## Figures and Tables

**Table 1:** Assignment of FT-IR bands [25,33,35,36].

Wavenumbers (cm^−1^)	Assignments
3680–3025	O–H stretch, H-bonded
2930	C–H stretch methyl and methylene groups (aliphatic)
2850	C–H stretch O–CH_3_ group
1716	C=O stretch, unconjugated ketone, carboxyl, and ester groups
1660	C=O stretch conj.
1600	Aromatic skeletal vibration
1510	Aromatic skeletal vibration
1455	CH deformations and aromatic ring vibrations
1420	Aromatic skeletal vibration combined with C–H in plane deformation
1368	Phenolic hydroxyl group
1325	Syringyl ring breathing, C–O stretch
1265	C–C, C–O, and C=O stretches in guaiacyl
1226	Guaiacyl ring breathing
1170	Aromatic C–H in plane deformation
1126	Syringyl ring breathing
1026	C–O–C
925	C–H deformation of out of plane, aromatic ring, pyranose ring
833	Aromatic CH out of plane deformation G + S

**Table 2:** Assignment of Main signals in the 2D HSQC NMR spectra of lignin [37].

Labels	δ_C_/δ_H_	Assignment
C_β_	52.81/3.55	C_β_−H_β_ in phenylcoumaran substructures (C)
B_β_	55.48/3.06	C_β_−H_β_ in resinol substructures (B)
−OCH_3_ (OMe)	55.48/3.58	C−H in methoxyls
A_γ_	59.83/3.63	C_γ_−H_γ_ in β-O-4 substructures (A)
I_γ_	62.08/4.13	C_γ_−H_γ_ in cinnamyl alcohol end- groups (I)
B_γ_	70.79/4.10,71.21/3.82	C_γ_−H_γ_ in resinol substructures (B)
B_a_	89.47/4.65	C_a_−H_a_ in resinol substructures (B)
A_β_	86.52/4.12	C_β_−H_β_ in β-O-4 substructures linked to a S unit (A)

## Data Availability

Not applicable.
